# ﻿Three new species and a new record of the genus *Lipolexis* (Hymenoptera, Braconidae, Aphidiinae) from South Korea

**DOI:** 10.3897/zookeys.1245.138802

**Published:** 2025-07-15

**Authors:** Sangjin Kim, JuHyeong Sohn, Hyojoong Kim

**Affiliations:** 1 Animal Systematics Laboratory, Department of Biology, Kunsan National University, Gunsan 54150, Republic of Korea Kunsan National University Gunsan Republic of Korea

**Keywords:** DNA barcoding, natural enemy, parasitoid wasps, systematics, taxonomy

## Abstract

The genus *Lipolexis* Förster, 1863 consists of 11 species worldwide, with two species previously recorded in South Korea. In this study, three new species: *L.depressiceps* S. Kim & H. Kim, **sp. nov.**, *L.sulcata* S. Kim & H. Kim, **sp. nov.**, and *L.longipetiolata* S. Kim & H. Kim, **sp. nov.** with an unrecorded species, *L.peregrina*, Tomanović & Kocić, 2020, are described and reported with photographs, mitochondrial cytochrome c oxidase subunit I (*COI*) data (barcode region), and a gene tree.

## ﻿Introduction

The genus *Lipolexis* Förster, 1863 (Hymenoptera: Braconidae: Aphidiinae) consists of 11 known species worldwide (Förster 1863; Gahan 1932; Chen 1981; Pramanik and Raychaudhuri 1984; Kocić et al. 2020), two of which have been already recorded in South Korea (Chang and Youn 1983; Starý and Choi 2000; NIBR 2023). In South Korea, Paik (1975) recorded *L.gracilis* Förster, 1863, and Chang and Youn (1983) recorded *L.oregmae* Gahan, 1932 (= *L.scutellaris* Mackauer, 1962). However, the evidence for *L.gracilis* is very limited, as the specimen used for identification is presumed lost, and the original record from Korea provides only limited description and figures, merely indicating its inclusion within the *gracilis* group. The record of *L.oregmae* also remains doubtful (pers. comm. Prof. Jong Cheol Paik, Mar. 2021) and further examination of both species is required. The forewing veins of *Lipolexis* are reduced (the m-cu and r-m veins are absent), and the vein RS is distinctly developed, like other genera in the subtribe Trioxina Ashmead, 1900. The ovipositor sheath is nearly straight but slightly bent downwards in the middle section (Chen and Shi 2001; Davidian 2007).

Most species have a limited distribution, except for three species: *L.gracilis*, *L.oregmae*, and *L.peregrina* Tomanović & Kocić, 2020, which are distributed in both Europe and Asia. *Lipolexisgracilis* is thought to be native to Europe, while *L.oregmae* is believed to have originated in Asia. Although *L.peregrina* requires further study, its phylogenetic position and the number of maxillary palp segments suggest that it also originated in Asia (Asian species have three maxillary palpomeres, while European species have four) (Kocić et al. 2020).

Based on morphological and molecular convergence, the genus *Lipolexis* can be subdivided into two large groups: the *gracilis* group and the *oregmae* group. These two groups have a genetic difference of more than 20%. Morphologically, the *gracilis* group has prominent central dorsal carinae of the petiole, while the *oregmae* group has a smooth dorsal petiole with prominent crenulated lateral longitudinal carinae. In addition, the *gracilis* group has a slightly shorter metacarpus (R1) than the *oregmae* group (the ratio of pterostigma 0.90–1.11× as long as metacarpus in the *gracilis* group, but 0.75–0.90× as long as in the *oregmae* group) (Kocić et al. 2020). The Asian species, *L.wuyiensis* Chen, 1981, *L.myzakkaiae* Pramanik & Raychaudhuri, 1984, and *L.pseudosutellaris* Pramanik & Raychaudhuri, 1984 are morphologically part of the *oregmae* group. Although these species are still regarded as valid, their descriptions and illustrations remain questionable (Lokeshwari et al. 2016; Kocić et al. 2020).

## ﻿Materials and methods

Holotypes of all new species have been deposited in the **NIBR** (National Institute of Biological Resources, Incheon) collection. Paratypes and other specimens are deposited in the **KSNU** (Kunsan National University, Gunsan 54150, Republic of Korea).

### ﻿Field and laboratory works

Samples were collected by using Malaise traps and sweeping with nets in various regions of South Korea between 2014 and 2023. The collected wasps were preserved in 80% ethanol at -19 °C.

The morphological and molecular identification of *Lipolexis* species are based on Kocić et al. (2020). We sorted specimens with similar phenotypes using a stereomicroscope (OLYMPUS SZX16, Leica M205C). After this initial sorting, we labeled the samples and proceeded with DNA extraction. Following morphological identification, we measured the specimens. For photography and characterisation, we used a LEICA DMC2900 digital camera mounted on a LEICA M205 C microscope (Leica Geosystems AG). Multiple images were taken at various focal heights using Mosaic V2.3 (Tucsen Software), and the image stacking process was carried out using HeliconFocus 7 (Helicon Soft) programs. After stacking, plates were generated using Adobe Photoshop CS6. To determine the precise shape of the specimens, we employed Mosaic V2.3 (Tucsen Software) (Berkovitch et al. 2009).

### ﻿Molecular analysis

Total genomic DNA was extracted using a LaboPass Tissue Kit (COSMOgenetech, Korea) following the manufacturer’s protocol with slight modifications. To preserve the morphological integrity of the specimens, we adapted the freezing method described by Yaakop et al. (2013). Our modification involved extending the incubation period from 30 min to 2 h at 56 °C with 200 μl of TL buffer and 20 μl of proteinase K. This adjustment allowed for effective DNA extraction while minimising damage to the specimens’ structure. Each sample underwent individual genomic DNA extraction to ensure sample-specific results.

For molecular identification, we targeted a 658-bp fragment from the front partial region of mitochondrial COI gene. This region was amplified using the primers, LCO1490 (forward) 5’-GGTCAACAAATCATAAAGATATTGG-3’ and HCO2198 (reverse) 5’-TAAACTTCAGGGTGACCAAAAAATCA-3’ (Folmer et al. 1994), with AccuPower PCR PreMix (Bioneer Corp., Daejeon, Korea). The polymerase chain reaction (PCR) was performed in a 20 μl reaction mixture consisting of 3 μl of DNA extract, 2 μl of primer, and 15 μl of ddH_2_O. The PCR protocol was carried out as follows: initial denaturation for 5 min at 95 °C, followed by 35 cycles of 60 s at 94 °C, 60 s at 54 °C, and 90 s at 72 °C, with a final extension at 72 °C for 7 min. PCR products were visualised on agarose gel electrophoresis. Visible bands were sent to Macrogen (Daejeon, Korea) for purification and sequencing analysis.

Sequence alignment was performed using MAFFT v. 7 (Katoh et al. 2019) with default settings, and frameshifts were checked to exclude pseudogenes. Sequence divergences were calculated using the ‘p-distance’ model with 1,000 bootstrap replications and pairwise deletion for data gaps in MEGA version 7.0 (Kumar et al. 2016).

A gene tree was inferred using the Bayesian method in BEAST2 (Bouckaert et al. 2014) and the Maximum Likelihood method in IQ-TREE (Trifinopoulos et al. 2016). To achieve accurate gene tree reconstruction, we performed best-fit substitution model testing in IQ-TREE (Trifinopoulos et al. 2016) under the GTR model, Corrected Akaike Information Criterion (AICc) for ML tree and the Bayesian Information Criterion (BIC) for BI tree. In BEAUti, we applied a strict clock model (Ferreira and Suchard 2008), with site options based on IQ-TREE results (GTR+F+G4). MCMC analysis was conducted and verified using Tracer (Rambaut et al. 2018) and DensiTree (Bouckaert 2010). Subsequently, we generated a consensus tree using TreeAnnotator with a posterior probability limit set to 1.0.

All sequences for analysis were processed using two efficient tools to define each species. ABGD (Automatic Barcode Gap Discovery) analysis was performed to automatically separate sequences into hypothetical molecular species by contrasting inter- and intra-specific distances (Puillandre et al. 2012) (https://bioinfo.mnhn.fr/abi/public/abgd/abgdweb.html). The Kimura K80 model was used with two relative gap widths (X = 1 and 1.5) in standard settings. Then, PTP (Poisson Tree Processes) analysis was conducted on the Exelixis Lab webserver (https://species.h-its.org/), using the maximum likelihood solution under standard settings (Zhang et al. 2013).

The 658 bp COI fragment sequenced for *Lipolexis* species was deposited in GenBank. For comparative analysis, we retrieved 43 sequences representing nine species, including an outgroup, from GenBank and BOLD (http://www.boldsystems.org) (Table 1).

**Table 1.** GenBank accession numbers of retrieved (1–11, 15, 17–22, 28–43) and newly generated molecular data (12–14, 16, 23–27) of species of *Lipolexis*.

No	Species	NCBI accession number	BOLD ID	References
1	*L.pakistanica*	KY832440	–	Kocić et al. (2020)
2	*L.pakistanica*	KY833346	–	Kocić et al. (2020)
3	*L.pakistanica*	KY831307	–	Kocić et al. (2020)
4	*L.pakistanica*	KY829945	–	Kocić et al. (2020)
5	*L.peregrina*	MF850280	–	Kocić et al. (2020)
6	*L.peregrina*	MF850281	–	Kocić et al. (2020)
7	*L.peregrina*	MF850282	–	Kocić et al. (2020)
8	*L.peregrina*	MW039064	–	Kocić et al. (2020)
9	*L.peregrina*	MW039067	–	Kocić et al. (2020)
10	*L.peregrina*	MW039065	–	Kocić et al. (2020)
11	*L.peregrina*	MW039066	–	Kocić et al. (2020)
12	*L.peregrina*	PV165855	–	In this study
13	*L.peregrina*	PV165856	–	In this study
14	*L.peregrina*	PV165857	–	In this study
15	*L.takadai*	–	GMCHN462–14	Kocić et al. (2020)
16	*L.depressiceps* sp. nov.	PV165858	–	In this study
17	*L.pelopsi*	KY887963	–	Kocić et al. (2020)
18	*L.pelopsi*	MW039050	–	Kocić et al. (2020)
19	*L.pelopsi*	MW039060	–	Kocić et al. (2020)
20	*L.pelopsi*	MW039061	–	Kocić et al. (2020)
21	*L.pelopsi*	MW039068	–	Kocić et al. (2020)
22	*L.pelopsi*	MW039072	–	Kocić et al. (2020)
23	*L.sulcata* sp. nov.	PV165859	–	In this study
24	*L.sulcata* sp. nov.	PV165860	–	In this study
25	*L.longipetiolata* sp. nov.	PV165861	–	In this study
26	*L.longipetiolata* sp. nov.	PV165862	–	In this study
27	*L.longipetiolata* sp. nov.	PV165863	–	In this study
28	*L.labialis*	MW039054	–	Kocić et al. (2020)
29	*L.labialis*	MW039056	–	Kocić et al. (2020)
30	*L.labialis*	MW039057	–	Kocić et al. (2020)
31	*L.labialis*	MW039058	–	Kocić et al. (2020)
32	*L.labialis*	MW039059	–	Kocić et al. (2020)
33	*L.labialis*	MW039069	–	Kocić et al. (2020)
34	*L.labialis*	MW039071	–	Kocić et al. (2020)
35	*L.gracilis*	–	GMBUC615–14	Kocić et al. (2020)
36	*L.gracilis*	–	GMBUA1116–14	Kocić et al. (2020)
37	*L.gracilis*	JN620635	–	Kocić et al. (2020)
38	*L.gracilis*	JN620636	–	Kocić et al. (2020)
39	*L.oregmaе*	EF207430	–	Kocić et al. (2020)
40	*L.oregmaе*	EF207431	–	Kocić et al. (2020)
41	*L.oregmaе*	–	GMBCM2962–15	Kocić et al. (2020)
42	*L.bengalensis*	–	GMBCI3219–15	Kocić et al. (2020)
43	*L.bengalensis*	–	GMBCI2841–15	Kocić et al. (2020)

## ﻿Results and discussion

**Figure 1.**
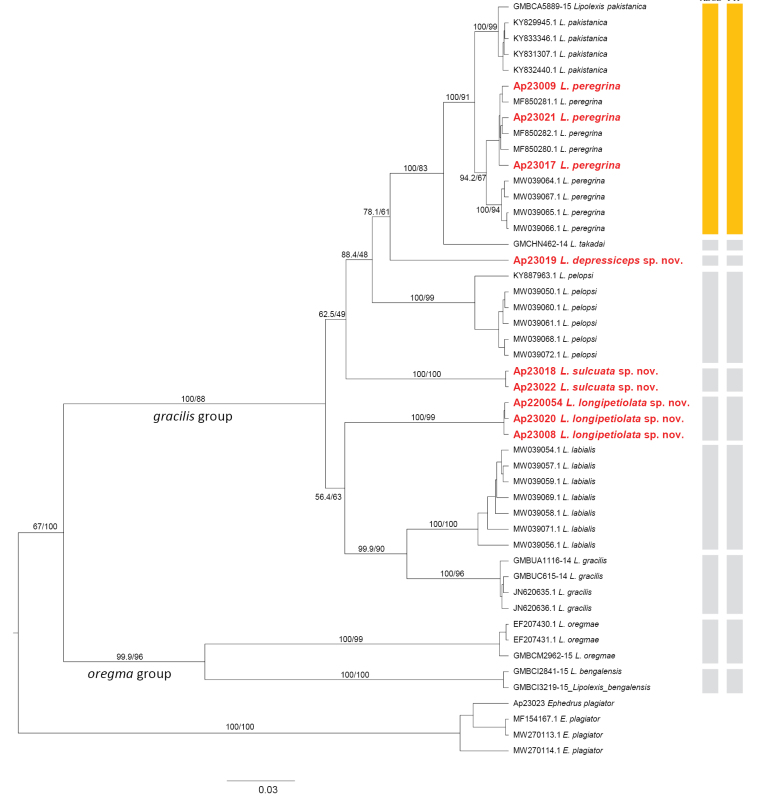
Gene tree of 11 *Lipolexis* spp. estimated by Bayesian and Maximum likelihood method using their COI DNA barcode data. *Ephedrusplagiator* was used as an outgroup. Bootstrap support values > 50% are indicated above branches. Scale bar indicates the expected rate of a nucleotide substitution.

A gene tree was constructed with 47 sequences from 12 species, including an outgroup. With robust support values, two main ingroups were identified: one is the *gracilis* group, and the other is the *oregmae* group (Fig. 1). Three new species: *Lipolexisdepressiceps* sp. nov., *L.sulcata* sp. nov., and *L.longipetiolata* sp. nov. were included in the *gracilis* group, with the previously unrecorded species *L.peregrina*. The *gracilis* group was further subdivided into two subgroups: *L.pakistanica* (emendation of *L.pakistanicus*), *L.peregrina* (emendation of *L.peregrinus*), *L.takadai* Tomanović & Kocić, 2020, *L.depressiceps* sp. nov., *L.pelopsi* Tomanović & Kocić, 2020, and *L.sulcata* sp. nov. clustered together, forming a subgroup closely related to the cluster (subgroup) comprising *L.depressiceps* sp. nov., *L.labialis*, and *L.gracilis* (Fig. 1).

Intraspecific and interspecific distances ranged from 0.000 to 0.025 (average 0.003) and from 0.023 to 0.210 (average 0.123), respectively (Table 2). The MOTUs of *L.peregrina* from South Korea formed a group with those from China (Fig. 1). In the first subgroup of the *gracilis* group, *L.depressiceps* sp. nov. is positioned between *L.takadai* and *L.pelopsi* (Fig. 1). The genetic distance between *L.depressiceps* sp. nov. and *L.takadai* is 0.082, while the distance to *L.pelopsi* is 0.088 (Table 2). Despite the morphologically significant traits, such as the number of maxillary palps, being more similar to *L.pelopsi* (*L.takadai* has three, while *L.depressiceps* sp. nov. and *L.pelopsi* each have four), the genetic position is closer to *L.takadai*. The genetic distance between *L.sulcata* sp. nov. and *L.pelopsi* is 0.084, which is slightly less than that of *L.depressiceps* sp. nov. (0.088) (Table 2). Despite all three species sharing four maxillary palpomeres, only *L.pelopsi* (which is positioned between the other two species in Fig. 1) has a pubescent mesoscutum with 18–20 setae and is found in the Mediterranean region (Kocić et al. 2020). *L.depressiceps* sp. nov. has nine or ten setae, *L.sulcata* sp. nov. has 6–8, and both are from Asia (South Korea).

**Table 2.** Calculated genetic distances based on COI sequences between *Lipolexis* species included in the analysis.

	*Lipolexispakistanica*	*L.peregrina*	*L.takadai*	*L.depressiceps*	*L.pelopsi*	*L.sulcata*	*L.longipetiolata*	*L.labialis*	*L.gracilis*	*L.oregmae*	*L.bengalensis*
	(*n* = 4)	(*n* = 10)			(*n* = 6)	(*n* = 2)	(*n* = 3)	(*n* = 7)	(*n* = 4)	(*n* = 3)	(*n* = 2)
*Lipolexispakistanica*	-0.003										
(*n* = 4)											
*L.peregrina*	0.023	-0.006									
(*n* = 10)											
*L.takadai*	0.045	0.039	0								
*L.depressiceps*	0.09	0.083	0.082	0							
*L.pelopsi*	0.081	0.066	0.064	0.088	-0.008						
(*n* = 6)											
*L.sulcata* (*n* = 2)	0.093	0.087	0.082	0.111	0.084	0					
*L.longipetiolata*	0.1	0.098	0.091	0.107	0.095	0.088	0				
(*n* = 3)											
*L.labialis*	0.122	0.115	0.1	0.115	0.089	0.101	0.107	-0.013			
(*n* = 7)											
*L.gracilis*	0.098	0.1	0.087	0.09	0.075	0.087	0.073	0.066	-0.001		
(*n* = 4)											
*L.oregmae*	0.19	0.184	0.179	0.198	0.191	0.21	0.183	0.202	0.183	-0.003	
(*n* = 3)											
*L.bengalensis*	0.2	0.201	0.198	0.192	0.195	0.192	0.201	0.196	0.187	0.174	0
(*n* = 2)											

In the second subgroup of the *gracilis* group, the species pair with the shortest genetic distance is *L.labialis* and *L.gracilis*, with a genetic distance of 0.066 (their genetic distances to *L.longipetiolata* sp. nov. are 0.107 and 0.073, respectively) (Table 2). These two European species have four maxillary palpomeres, whereas *L.longipetiolata* sp. nov., from South Korea, has three maxillary palpomeres. Interestingly, the branching pattern differs between the first and second subgroups with respect to the number of maxillary palpomeres (Fig. 1). In the first subgroup, the species with four maxillary palpomeres were placed basally, while those with three occur in more derived positions. In the second subgroup, the species with three maxillary palpomeres appear earlier in the topology than those with four (Fig. 1).

The difference in the number of maxillary palpomeres is considered a distinguishing feature between species of Asian and European origin. Although Asian species typically have three segments and European species have four maxillary palpomeres, the two newly reported species from South Korea, *L.depressiceps* sp. nov. and *L.sternaulata* sp. nov., both have four palpomeres. Further research based on additional specimens is needed to determine whether the number of palpomeres is a characteristic related to the species’ origin. Within the *gracilis* group, the genetic difference was 0.098 (0.075–0.122), and the group was subdivided into two smaller subgroups. However, since all specimens were collected using a Malaise trap, it could not be confirmed whether they are clustered based on different host associations in this study.

In the ABGD and PTP analyses, *L.pakistanica* and *L.peregrina* are considered the same species (Fig. 1). Despite the low genetic distance values, they exhibit clear morphological differences according to the original description (Kocić et al. 2020), including differences in the length/width ratios of F1 and F2, pterostigma length/width, petiole length/width, and ovipositor sheath length/width. According to the authors of both analysis methods (Puillandre et al. 2012; Zhang et al. 2013), these results are only species estimates and should be complemented with other evidence in an integrative taxonomic approach.

### ﻿Systematic accounts (description on the basis of females)

#### ﻿Genus Lipolexis

Taxon classificationAnimaliaHymenopteraBraconidae

Förster, 1863

BCD4A887-58CA-5CFF-BB04-2FAB85C26361

Gynocryptus Quilis, 1931: 25–30.

##### Type species.

*Lipolexisgracilis* Förster, 1863, type locality Germany.

#### ﻿ Lipolexis depressiceps

Taxon classificationAnimaliaHymenopteraBraconidae

S. Kim & H. Kim sp. nov.

8AC11699-4351-57C5-B8AA-138EB7CD128B

https://zoobank.org/20496CCF-AFDF-4054-AAFE-44EAAAFF5189

Figs 2A–M , 6E

##### Type material.

***Holotype***: South Korea • ♀; 1549, Chusan-ri, Ongnyong-myeon, Gwangyang-si, Jeollanam-do; 35°01.8'N, 126°36'E; 10–24 Sep. 2019 by Malaise trap; leg. S. Kim; GenBank: PV165858 (Ap23019).

##### Diagnosis.

In some morphological characters (number of antennal segments, maxillary and labial palpomeres, central carinae of dorsal petiole), *Lipolexisdepressiceps* sp. nov. is similar to the other three *gracilis* group species, *L.gracilis*, *L.pelopsi*, and *L.sulcata* sp. nov.. However, it clearly differs from them in having deeply depressed and anteriorly narrowed occipital carina (partially obscured in dorsal aspect, while distinctly visible in other species), distinctly elongated F1 and F2 (length/width ratio = 4.7 and 4.3, while < 4.0 in other species), shorter pterostigma/R1 ratio (0.8, while 1.0–1.1 in *L.gracilis*, 0.9–1.1 in *L.pelopsi* and 1.1 in *L.sulcata* sp. nov.), and more elongated hind femur (length/width = 5.2, while < 4.5 in other species).

##### Description.

**Figure 2.**
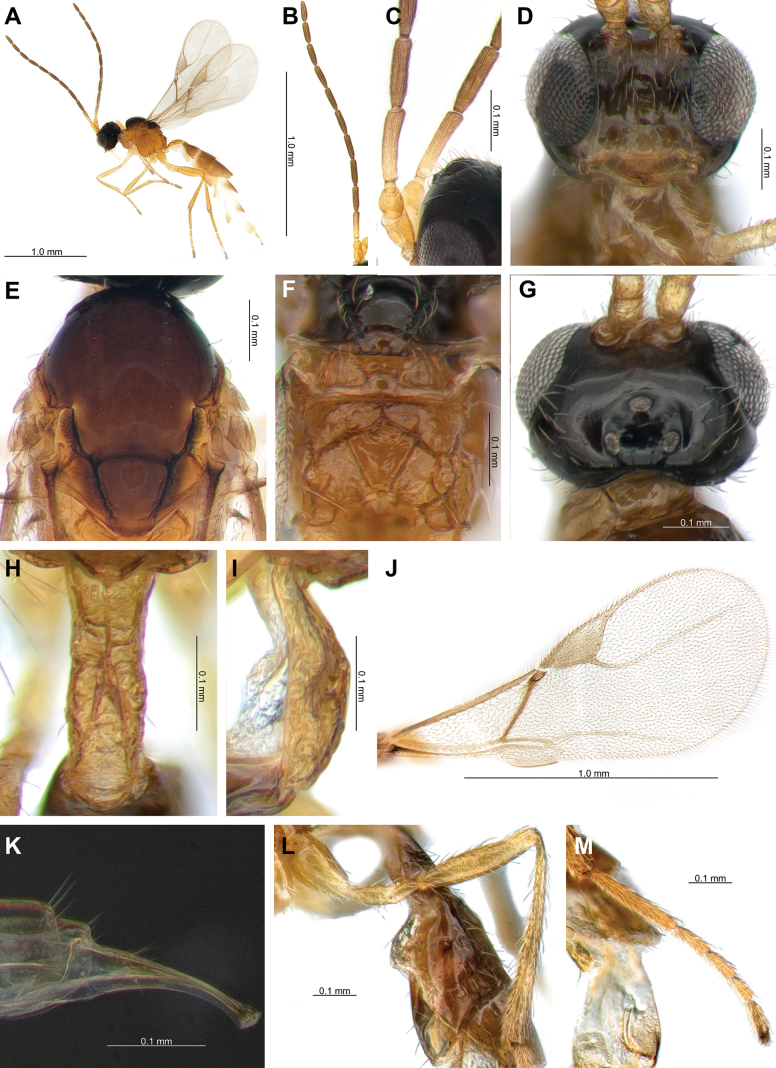
*Lipolexisdepressiceps* sp. nov. **A.** Habitus; **B.** Antenna; **C.** F1 and F2; **D.** Head; **E.** Mesoscutum; **F.** Propodeum; **G.** Dorsal aspect of head; **H.** Dorsal aspect of petiole; **I.** Lateral aspect of petiole; **J.** Forewing; **K.** Ovipositor; **L.** Hind femur and tibia; **M.** Hind tarsus.

**Female.** Length of body about 3.0 mm (Fig. 2A). Length of forewing 1.5 mm (Fig. 2J).

***Head*.** Width of head 1.5× its maximum length in dorsal aspect; occipital carina narrow and deeply compressed anteriorly; head 1.3× wider than mesosoma, with sparse long setae; eye 1.7× as long as temple in dorsal aspect; ocello-ocular line (OOL) 3.1× as long as posterior (= lateral) ocellus diameter; ratio of OOL: antero-posterior ocellar line (AOL): postero-ocellar line (POL) = 25: 10: 14 (Fig. 2G). Eyes oval, sparsely setose; face with densely long setae; width/height ratio = 1.1; tentorial index = 0.4; clypeus oval with 12 long setae; malar space 0.1× as long as longitudinal eye diameter (Fig. 2D). Antenna 12-segmented; F1 equal to F2; F1 and F2 4.7× and 4.3× as long as their width at the middle, respectively; F1 with three longitudinal placodes and F2 with four longitudinal placodes (Fig. 2B, D). Maxillary palp with four palpomeres, labial palp with one palpomere.

***Mesosoma*.** Mesoscutum with notaulices on anterolateral margin, effaced dorsally (Figs 2E, 6E). Dorsal surface smooth, with two rows of 9–10 long setae along the dorsolateral part of mesoscutum; scutellum nearly triangular, with 2 long setae on each side (Fig. 2E). The precoxal sulcus is weakly impressed on the anterior part of the mesopleuron (Fig. 6E). Propodeum areolated, areola length/width ratio = 1.1 (Fig. 2F). Pterostigma 2.4× as long as width; pterostigma 0.8× as long as vein R1 (= metacarpus); r and RS vein extended (Fig. 2J).

***Leg*.** Hind femur slender, length/width ratio = 5.2; hind tibia length/width ratio = 11.6; hind femur: hind tibia: hind tarsus = 1: 1.6: 1.8 (Fig. 2L, K).

***Metasoma*.** Petiole elongated, 3.1× as long as wide at spiracles; distinctly prominent central carinae, situated along the dorsal surface of the petiole, start at the anterior part and bifurcate near spiracles; dorsolateral part of petiole posterior of spiracles concave on each side (Fig. 2H, I). Ovipositor sheath slender and long, wide at base, curved downwards; ratio of ovipositor sheath length/width 3.1 at base and 8.8 at tip (Fig. 2K).

***Colour*.** Antenna brown; scape and pedicel yellowish brown, F1 at least partly yellowish brown. Head and face black, clypeus with mouthparts yellowish brown. Mesosoma and metasoma brown; mesoscutum black; petiole brown. Legs pale brown with dark apices.

##### Distribution.

South Korea.

##### Etymology.

*Lipolexisdepressiceps* sp. nov. is derived from the Latin words *depressus* (meaning pressed down or flattened) and *ceps* (meaning ‘head’), referring to its distinctly anteriorly depressed vertex in dorsal aspect.

#### ﻿ Lipolexis sulcata

Taxon classificationAnimaliaHymenopteraBraconidae

S. Kim & H. Kim sp. nov.

DD04DE8B-9114-5479-818F-ECA4AFFBB295

https://zoobank.org/3824E14E-3998-42F1-8692-B731AF283E8E

Figs 3A–M , 6F

##### Type material.

***Holotype***: South Korea • ♀; 1549, Chusan-ri, Ongnyong-myeon, Gwangyang-si, Jeollanam-do; 35°01.8'N, 126°36'E; 12–27 Oct. 2019 by malais trap; leg. S. Kim; GenBank: PV165859 (Ap23018). ***Paratype***: South korea • 1♀; same location as for holotype; 24 Sep. – 08 Oct. 2019 by Malaise trap; leg. S. Kim; GenBank: PV165860 (Ap23022).

##### Diagnosis.

In some morphological characters (number of antennal segments, maxillary palps, and labial palps, central carinae of dorsal petiole), *Lipolexissternaulata* sp. nov. is similar to the other three *gracilis* group species, *L.gracilis*, *L.pelopsi*, and *L.depressiceps* sp. nov. However, it clearly differs from them in having distinctly developed sternaulus (= precoxal sulcus) on the anterior part of the mesopleuron, not reaching the metapleuron (while weakly impressed in other species), shorter tibia than *L.gracilis* and *L.pelopsi* (length/maximum length ratio = 8.6, while 14.0 and 17.4 in *L.gracilis* & *L.pelopsi*), elongated ovipositor sheath (length/ width ratio = 3.2–4.0 at base and 9.7–10.6 at tip, while 3.1 at base and 9.1 at tip in *L.pelopsi*, 2.9 at base and 9.6 at tip in *L.gracilis* and 3.1 at base and 8.8 at tip in *L.depressiceps* sp. nov.), and elongated pterostigma (length/width ratio= 2.6–2.7, while 2.3–2.6 in other species).

##### Description.

**Figure 3.**
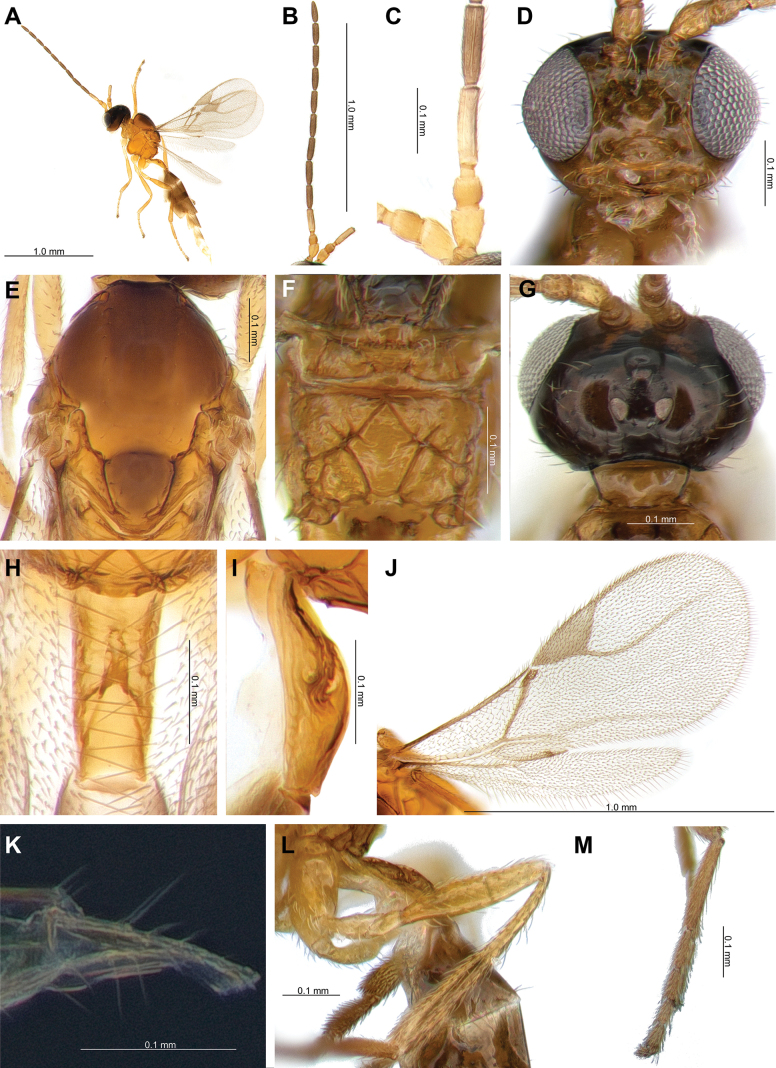
*Lipolexissulcata* sp. nov. **A.** Habitus; **B.** Antenna; **C.** F1 and F2; **D.** Head; **E.** Mesoscutum; **F.** Propodeum; **G.** Dorsal aspect of head; **H.** Dorsal aspect of petiole; **I.** Lateral aspect of petiole; **J.** Forewing; **K.** Ovipositor; **L.** Hind femur and tibia; **M.** Hind tarsus.

**Female.** Length of body about 2.4 mm (Fig. 3A). Length of forewing 1.3 mm (Fig. 3K).

***Head*.** Width of head 1.5× its maximum length in dorsal aspect; occipital carina gently concave; head 1.3× wider than mesosoma, with sparse long setae; eye 1.6× as long as temple in dorsal aspect; OOL 3.1× as long as posterior ocellus diameter; ratio of OOL: AOL: POL = 26: 10: 10 (Fig. 3G). Eyes oval, sparsely setose; face with sparse long setae; width/height ratio= 1.2; densely long setae around antennal socket; tentorial index= 0.3; clypeus oval and with 7 long setae; malar space 0.2× as long as longitudinal eye diameter (Fig. 3D). Antenna 12-segmented ; F1 equal to F2; F1 and F2 3.4–3.6× and 3.5–3.8× as long as their width at the middle, respectively; F1 and F2 with three longitudinal placodes (Fig. 3B, C). Maxillary palp with four palpomeres, labial palp with one palpomere.

***Mesosoma*.** Mesoscutum with notaulices on anterolateral margin, effaced dorsally (Figs 3E, 6F). Dorsal surface smooth, with two rows of 6–8 long setae along the dorsolateral part of mesoscutum, respectively; scutellum nearly triangular, with three and four long setae on each side (Fig. 3E). The precoxal sulcus is present on the anterior part of the mesopleuron, not reaching the metapleuron (Fig. 6F). Propodeum areolated, areola length/width ratio= 1.3 (Fig. 3F). Pterostigma 2.6–2.7× as long as width; Pterostigma 1.1× as long as vein R1 (= metacarpus); r and RS vein extended (Fig. 3J).

***Leg*.** Hind femur length/width ratio = 4.3; hind tibia length/width ratio = 11.3; hind femur: hind tibia: hind tarsus = 1: 1.6: 1.7 (Fig. 3L, M).

***Metasoma*.** Petiole elongated, wide at base, slightly narrowing towards the apex; 3.1–3.3× as long as wide at spiracles; distinctly prominent central carinae, situated along the dorsal surface of the petiole, and bifurcate near of spiracles; dorsolateral part of posterior spiracles is concave on each side (Fig. 3H, I). Ovipositor sheath elongated, wide at base, curved downwards; ratio of ovipositor sheath length/width = 3.2–4.0 at base and 9.7–10.6 at tip (Fig. 3K).

***Colour*.** Antenna brown; scape and pedicel yellowish brown, F1 yellowish brown at least basal 1/3 partly yellowish brown, gradually brown to apex. Head, face and clypeus with mouthparts pale brown. Mesosoma pale brown and metasoma brown; mesoscutum brown; petiole pale brown. Legs pale brown with dark apices.

##### Distribution.

South Korea.

##### Etymology.

*Lipolexissternaulata* sp. nov. is derived from the Latinised form of the precoxal sulcus, referring to its distinctly developed precoxal sulcus on the mesopleuron.

#### ﻿ Lipolexis longipetiolata

Taxon classificationAnimaliaHymenopteraBraconidae

S. Kim & H. Kim sp. nov.

9A82AD3B-DA04-58AA-9342-C7D09BF4E667

https://zoobank.org/CAF62962-B08E-4C4C-824C-AA26693A84C9

Figs 4A–M , 6G

##### Type material.

***Holotype***: South Korea • ♀; 434, Buun-ri, Sannae-myeon, Namwon-si, Jeonbuk-do; 35°23'N, 127°34'E; 09 Jun. 2022; leg. S. Kim; GenBank: PV165863 (Ap220054). ***Paratype***: South Korea • 1♀; 1549, Chusan-ri, Ongnyong-myeon, Gwangyang-si, Jeollanam-do; 35°01.8'N, 126°36'E; 24 Sep.–08 Oct. 2019 by malaise trap; leg. S. Kim; GenBank: PV165862 (AP23020) • 1♀; 716, Donggang-ro, Yeongwol-eup, Yeongwol-gun, Gangwon-do; 37°13.9'N, 128°30'E; 04 – 23 Jun. 2014 by malaise trap; leg. S. Kim; GenBank: PV165861 (Ap23008).

##### Diagnosis.

In some morphological characters (number of antennal segments, maxillary and labial palpomeres, central dorsal carinae of petiole), *Lipolexislongipetiolata* sp. nov. is similar to other three *gracilis* group species, *L.peregrina*, *L.takadai*, and *L.pakistanica*. However, it clearly differs from them in having elongated petiole (petiole length/width ratio = 3.4–3.6, while 3.1–3.3 in *L.peregrina*, 2.8 in *L.takadai*, and 2.7 in *L.pakistanica*), elongated F1 than *L.peregrina* (length/width ratio = 4.3, while 3.1–3.8 in *L.peregrina*), stouter tibia (length/maximum width ratio = 8.9, while 11.0 in *L.peregrina*), and smaller ocellus (OOL/posterior ocellus diameter ratio= 5.0, while 3.0 in *L.peregrina*).

##### Description.

**Figure 4.**
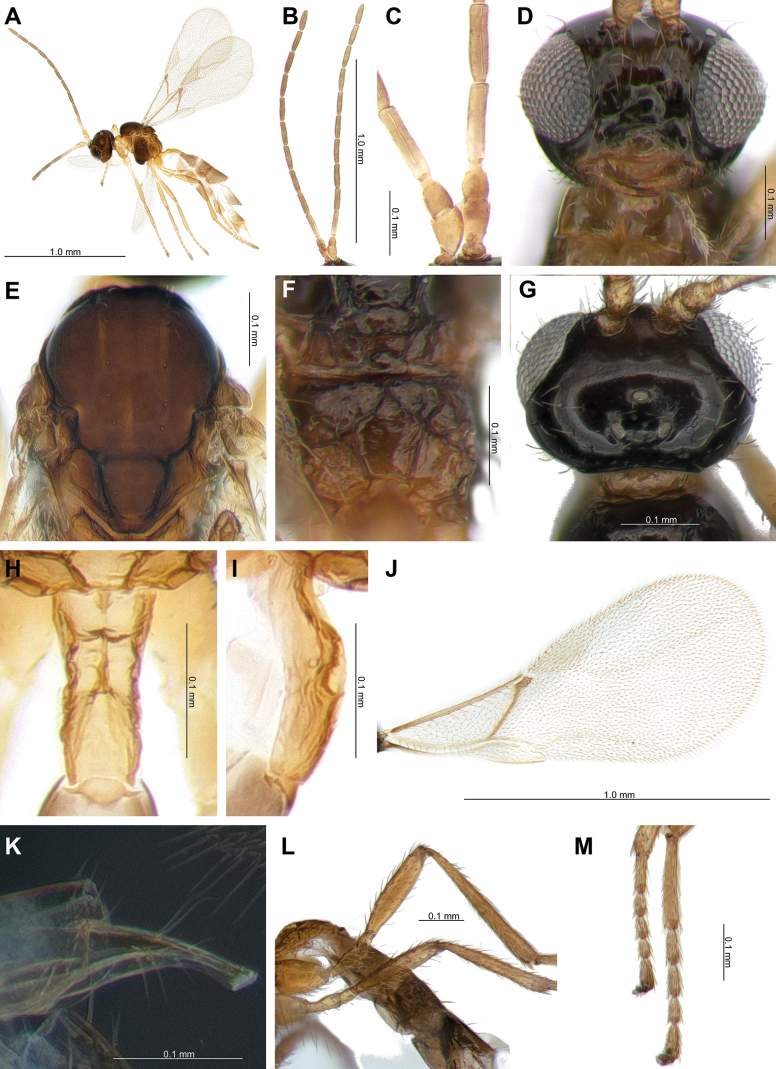
*Lipolexislongipetiolata* sp. nov. **A.** Habitus; **B.** Antenna; **C.** F1 and F2; **D.** Head; **E.** Mesoscutum; **F.** Propodeum; **G.** Dorsal aspect of head; **H.** Dorsal aspect of petiole; **I.** Lateral aspect of petiole; **J.** Forewing; **K.** Ovipositor; **L.** Hind femur and tibia; **M.** Hind tarsus.

**Female.** Length of body about 2.1 mm (Fig. 4A). Length of forewing 1.2 mm (Fig. 4J).

***Head*.** Width of head 1.4× its maximum length in dorsal aspect; occipital carina gently concave; head 1.3× wider that mesosoma, with sparse long setae; eye 1.5× as long as temple in dorsal aspect; OOL 5.0× as long as posterior ocellus diameter; ratio of OOL: AOL: POL = 29: 10: 10 (Fig. 4G). Eyes oval, sparsely setose; face with sparse long setae; width/height ratio = 1.1; tentorial index = 0.4; clypeus oval with 8 long setae; malar space 0.2× as long as longitudinal eye diameter (Fig. 4D). Antenna 12-segmented; F1 equal or longer than F2 (1.0–1.1); F1 and F2 4.3× and 3.8–4.2× as long as their width at the middle, respectively; F1 and F2 with two longitudinal placodes respectively (Fig. 4B, C). Maxillary palp with three palpomeres, labial palp with one palpomere.

***Mesosoma*.** Mesoscutum with notaulices on anterolateral margin, effaced dorsally (Figs 4E, 6G). Dorsal surface smooth, with two rows of six long setae along the dorsolateral part of mesoscutum; scutellum nearly triangular, with three long setae on each (Fig. 4E). The precoxal sulcus is weakly impressed on the anterior part of the mesopleuron (Fig. 6G). Propodeum areolated, areola length/width ratio = 1.1 (Fig. 4F). Pterostigma 2.6–3.0× as long as width; pterostigma 0.8× as long as vein R1 (= metacarpus); r and RS vein extended (Fig. 4J).

***Leg*.** Hind femur length/width ratio= 4.5; hind tibia length/width ratio = 8.6; hind femur: hind tibia: hind tarsus = 1: 1.5: 1.8 (Fig. 4M, L).

***Metasoma*.** Petiole long and slender, wide at base, slightly narrowing towards the apex. 3.4–3.6× as long as wide at spiracles; distinctly prominent central carinae, situated along the dorsal surface of the petiole, start at the anterior part and bifurcate near spiracles; dorsolateral part of petiole posterior of spiracles concave on each side (Fig. 4H, I). Ovipositor sheath slender and long, wide at base, curved downwards; ratio of ovipositor sheath width/length = 3.5–4.0 at base and 8.3–8.4 at tip (Fig. 4K).

***Colour*.** Antenna brown; scape and pedicel pale brown, F1 pale brown at least basal 1/3 partly yellowish brown, gradually pale brown to apex. Head and face dark brown, clypeus with mouthparts pale brown. Mesosoma dark brown and metasoma brown; mesoscutum dark brown; petiole pale brown. Legs pale brown with dark apices.

##### Distribution.

South Korea.

##### Etymology.

*Lipolexislongipetiolata* sp. nov. derived from the Latin words *longus* (meaning long) and *petioles* (meaning petiole), referring to its distinctly elongated petiole.

#### ﻿ Lipolexis peregrina

Taxon classificationAnimaliaHymenopteraBraconidae

Tomanović & Kocić, 2020

8228C8F2-7947-581A-B32E-172B0A60521B

Figs 5A–M , 6H

Lipolexis peregrinus Tomanović & Kocić, 2020: 667.

##### Specimen examined.

South Korea • 1♀; 666-53, Miwon-ri, Miwon-myeon, Sangdang-gu, Cheongju-si, Chungcheongbuk-do; 36°37'N, 127°39'E; 30 Jun. – 15 Jul. 2015. by malais trap; leg. S. Kim; GenBank: PV165855 (Ap23009) • 1♀; 852, Hyanggyo-ri, Cheongung-myeon, Imsil-gun, Jeonbuk-do; 35°34.9'N, 127°12'E; 07 Jun. 2019; leg. S. Kim; GenBank: PV165857 (AP23017) •1♀; 1549, Chusan-ri, Ongnyong-myeon, Gwangyang-si, Jeollanam-do; 35°01.8'N, 126°36'E; 24 Sep.– 08 Oct. 2019 by malais trap; leg. S. Kim; GenBank: PV165856 (Ap23021).

##### Redescription.

**Figure 5.**
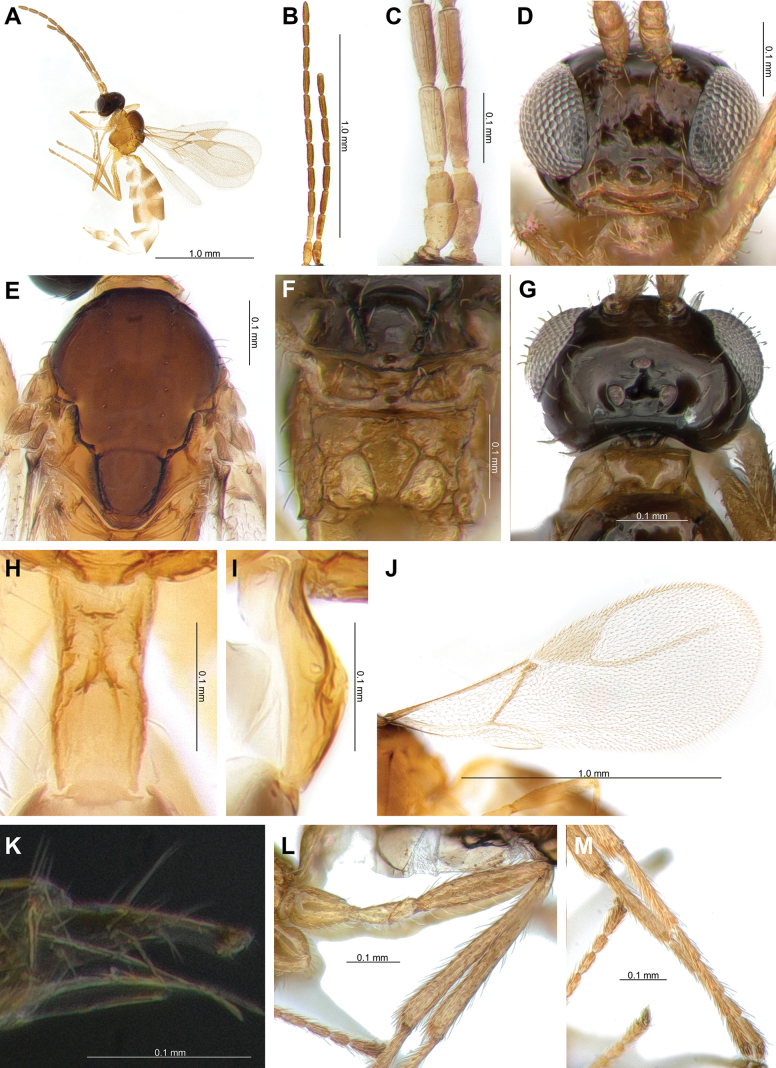
*Lipolexisperegrina* Tomanović & Kocić, 2020. **A.** Habitus; **B.** Antenna; **C.** F1 and F2; **D.** Head; **E.** Mesoscutum; **F.** Propodeum; **G.** Dorsal aspect of head; **H.** Dorsal aspect of petiole; **I.** Lateral aspect of petiole; **J.** Forewing; **K.** Ovipositor; **L.** Hind femur and tibia; **M.** Hind tarsus.

**Female.** Length of body about 2.1mm (Fig. 5A). Length of forewing 1.3 mm (Fig. 5J).

**Figure 6.**
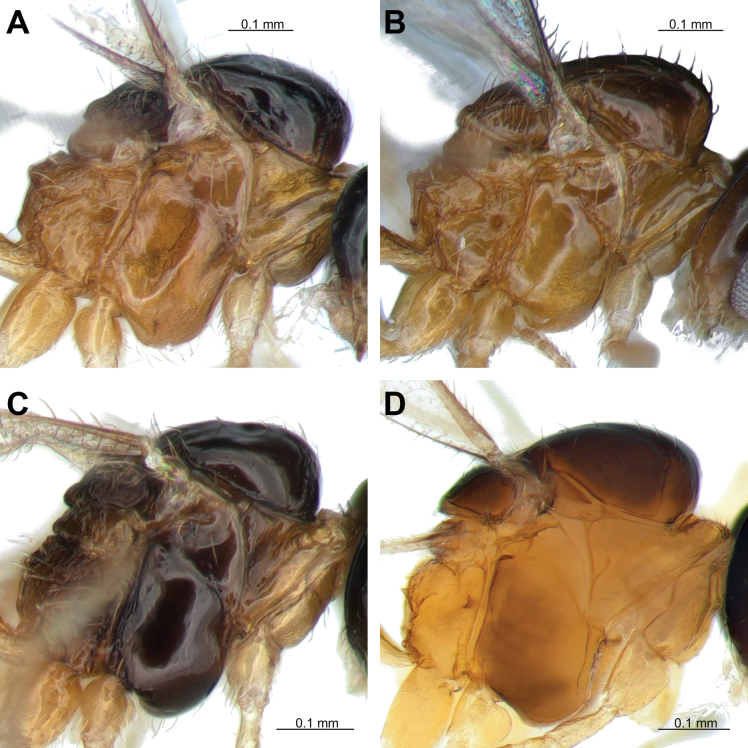
Lateral aspect of mesosoma. **A.***Lipolexisdepressiceps* sp. nov.; **B.***L.sulcata* sp. nov.; **C.***L.longipetiolata* sp. nov.; **D.***L.peregrina*.

***Head*.** Width of head 1.5× its maximum length in dorsal aspect; occipital carina broad and moderately concave anteriorly; maximum head length 1.3× as long as its minimum length; head 1.2× wider that mesosoma, with sparse long setae; eye 1.7× as long as temple in dorsal aspect; OOL 3.0× as long as posterior ocellus diameter; ratio of OOL: AOL: POL = 17: 10: 7 (Fig. 5G). Eyes oval, sparsely setose; face densely long setose, width/height ratio = 1.3; tentorial index = 0.32; clypeus oval and with 8 setae; malar space 0.2× as long as longitudinal eye diameter (Fig. 5D). Antenna 12-segmented; F1 subequal to F2 (F2 0.9 to 1.1× as long as F1); F1 and F2 3.1–3.8× and 3.4–3.6× as long as their width at the middle, respectively; F1 and F2 with two and three longitudinal placodes, respectively (Fig. 5B, C). Maxillary palp with three palpomeres, labial palp with one palpomere.

***Mesosoma*.** Mesoscutum with notauli on anterolateral margin, effaced dorsally; The precoxal sulcus is weakly impressed on the anterior part of the mesopleuron (Figs 5E, 6H). Dorsal surface smooth, with two rows of four or five long setae along the dorsolateral part of mesoscutum; scutellum nearly triangular, with two long setae on each side (Fig. 5E). Propodeum areolated, areola length/width ratio = 1.2 (Fig. 5F). Pterostigma 2.5–2.7× as long as width; pterostigma 0.8–0.9× as long as vein R1 (= metacarpus); r and RS vein extended (Fig. 5J).

***Leg*.** Hind femur length/width ratio= 5.0; hind tibia length/width ratio = 11.0; hind femur: hind tibia: hind tarsus = 1: 1.6: 1.7 (Fig. 5L, M).

***Metasoma*.** Petiole elongated, 3.1–3.3× as long as wide at spiracles; distinctly prominent central carinae, situated along the dorsal surface of the petiole, start at the anterior part and bifurcate near spiracles; dorsolateral part of posterior spiracles concave on each side (Fig. 5H, I). Ovipositor sheath slender and long, wide at base, curved downwards; ratio of ovipositor sheath width/length 3.0 (2.9–3.1) at base and 8.5 (7.9–9.3) at tip (Fig. 5K).

***Colour*.** Antenna brown; scape and pedicel brown, F1 entirely or at least partly pale brown. Head and face dark brown, clypeus with mouthparts pale brown. Mesosoma and metasoma brown; mesoscutum dark brown; petiole pale brown. Legs pale brown with dark apices.

##### Distribution.

Europe (Spain and Slovenia) and Oriental region (China, Japan [from GenBank], and South Korea).

### ﻿Key to Korean species of *Lipolexis*

The record of *Lipolexisoregmae* from South Korea is doubtful (pers. comm. Prof. Jong Cheol Paik); evidence of *L.gracilis* from South Korea is limited, a European species (Kocić et al. 2020).

**Table:** 

1	Petiole dorsally smooth, bearing crenulated lateral longitudinal carinae (*oregmae* group); maxillary palps with 3 palpomeres, labial palps with 1 palpomere; F2 is 4× as long as wide; number of longitudinal placodes on F1 and F2, 2–3 and 4–5, respectively	***Lipolexisoregmae***
–	Petiole with prominent bifurcating central carina dorsally, without crenulated lateral longitudinal carinae (Figs 2H, 3H, 4H, 5H) (*gracilis* group)	**2**
2	Maxillary palps with 4 palpomeres	**3**
–	Maxillary palps with 3 palpomeres	**5**
3	Occipital carina narrow and deeply compressed anteriorly, partially obscured in dorsal aspect (Fig. 2G); F1 is 4.7× as long as wide (Fig. 2C); hind femur is 5.2× as long as wide (Fig. 2L); ovipositor sheath is 8.8× as long as wide at tip (Fig. 2K)	***Lipolexisdepressiceps* sp. nov.**
–	Occipital carina weakly concaved, distinctly visible in dorsal aspect (Fig. 3G; Suppl. material 1: fig. 1A); Fl is <4.0× as long as wide (Fig. 3C); hind femur is < 4.5× as long as wide (Fig. 3L); ovipositor sheath is >9.5× as long as wide at tip (Fig. 3K)	**4**
4	Distinctly developed precoxal sulcus on anterior part of mesopleuron, not reaching metapleuron (Figs 3A, 6F); ocello-ocular line (OOL) is 3.1× as long as maximum length of posterior (=lateral) ocellus (Fig. 3G); hind femur is 4.3× as long as wide (Fig. 3L); hind tibia is 11.3× as long as wide (Fig. 3L); pterostigma is >2.6× as long as wide (Fig. 3J); ovipositor sheath is 3.2–4.0× as long as wide at base, 9.7–10.6 × as long as wide at tip (Fig. 3K)	***Lipolexissulcata* sp. nov.**
–	Weakly impressed precoxal sulcus on anterior part of mesopleuron (Fig. 6A; Suppl. material 1: fig. 1B); OOL is 3.6× as long as maximum length of posterior ocellus (Suppl. material 1: fig. 1A); hind femur is 4.0× as long as wide; hind tibia 14.0× as long as wide; pterostigma is <2.5× as long as wide; ovipositor sheath 2.9× as long as wide at base, 9.6× as long as wide at tip	***Lipolexisgracilis***
5	F1 is 4.3× as long as wide (Fig. 4C); occipital carina weakly concaved, almost straight in dorsal aspect (Fig. 4G); OOL is 5.0× as long as maximum length of posterior ocellus (Fig. 4G); hind femur is 4.5× as long as wide (Fig. 4L); hind tibia is 8.6× as long as wide (Fig. 4L); petiole is 3.4–3.6× as long as wide (Fig. 4H, I); ovipositor sheath is 3.5–4.0× as long as wide at base, 8.3–8.4 at tip (Fig. 4J)	***Lipolexislongipetiolata* sp. nov.**
–	F1 is 3.1–3.3× as long as wide (Fig. 5C); occipital carina broad and deeply concaved anteriorly in dorsal aspect (Fig. 5G); OOL is 3.0× as long as maximum length of posterior ocellus (Fig. 5G); hind femur length is 5.0× as long as wide (Fig. 5L); hind tibia is 11.0× as long as wide (Fig. 5L); petiole is <3.3× as long as wide (Fig. 5H, I); ovipositor sheath is 2.9–3.1× as long as wide at base, 7.9–9.3× as long as wide at tip (Fig. 5K)	***Lipolexisperegrina***

## Supplementary Material

XML Treatment for Lipolexis

XML Treatment for Lipolexis depressiceps

XML Treatment for Lipolexis sulcata

XML Treatment for Lipolexis longipetiolata

XML Treatment for Lipolexis peregrina

## ﻿Additional information

### Use of AI

No use of AI was reported.

### Author ORCIDs

Sangjin Kim https://orcid.org/0000-0001-8395-8019

JuHyeong Sohn https://orcid.org/0000-0003-0976-4114

Hyojoong Kim https://orcid.org/0000-0002-1706-2991

### Data availability

All of the data that support the findings of this study are available in the main text or Supplementary Information.

## Supplementary materials

Supplementary material 1 Supplementary figure Data type tif Explanation note *Lipolexisgracilis* Förster, 1863. **A.** Dorsal aspect of head; **B.** Lateral aspect of mesosoma. https://binary.pensoft.net/file/1374426This dataset is made available under the Open Database License (http://opendatacommons.org/licenses/odbl/1.0/). The Open Database License (ODbL) is a license agreement intended to allow users to freely share, modify, and use this Dataset while maintaining this same freedom for others, provided that the original source and author(s) are credited.Sangjin Kim, JuHyeong Sohn, Hyojoong Kim
