# Supplementary figures and images for: ﻿Three new species and a new record of the genus Lipolexis (Hymenoptera, Braconidae, Aphidiinae) from South Korea
Source: Zookeys. 2025 Jul 15;1245:323–42. doi: 10.3897/zookeys.1245.138802 (PMC12284590; doi:10.3897/zookeys.1245.138802)

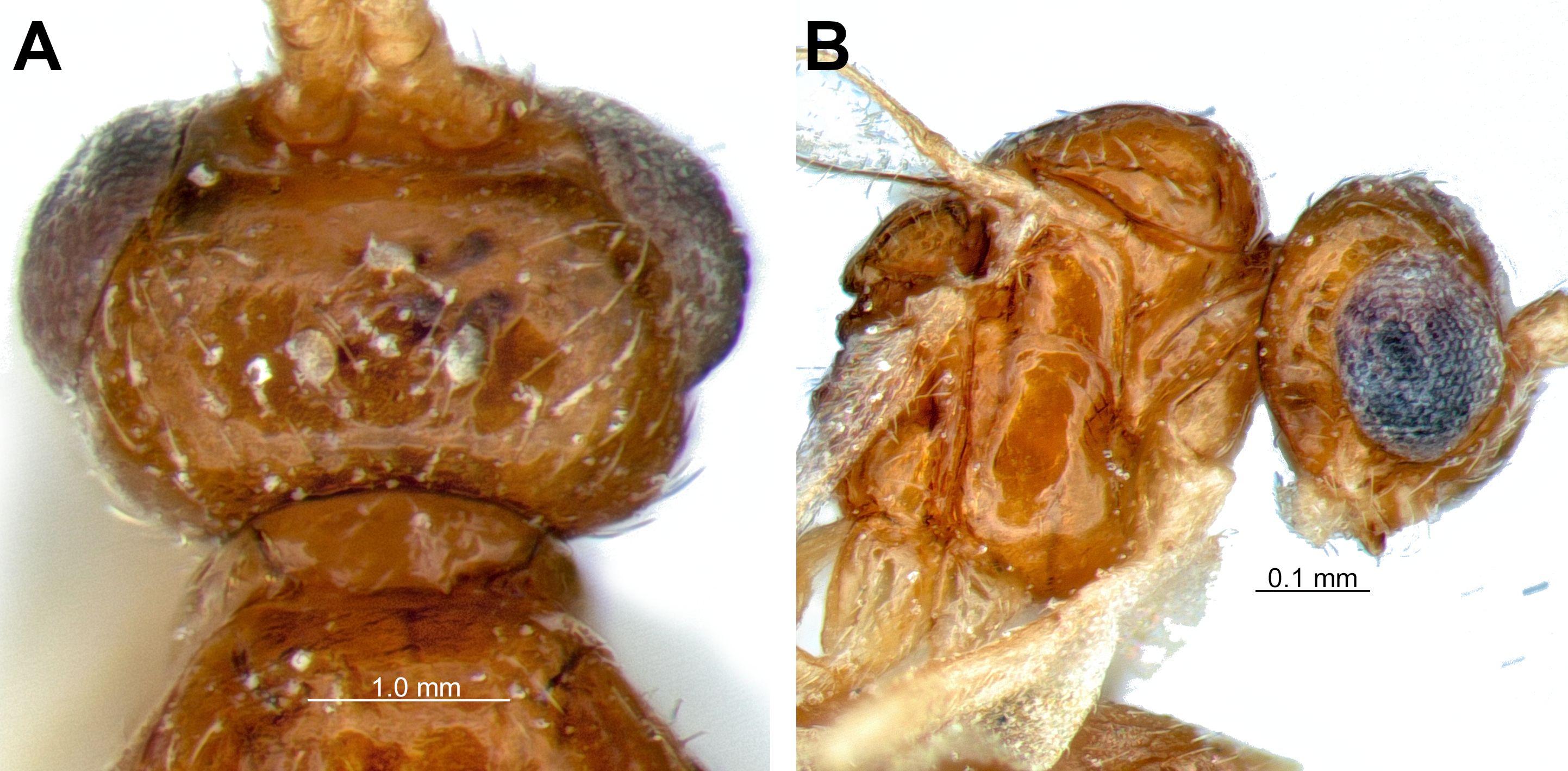

Supplement: Supplementary material 1 — Supplementary figure [file zookeys-1245-323_article-138802__-s001.tif]
